# Patterns of court-ordered behavioral health evaluations among extreme risk protection order respondents in Washington State

**DOI:** 10.1186/s40621-026-00698-x

**Published:** 2026-07-10

**Authors:** Rachel Ross, Erika Marts, Nicole Asa, Samantha Banks, Kelsey M. Conrick, Esprene Liddell-Quintyn, Ayah Mustafa, Sandra B. Shanahan, Julia P. Schleimer, Kimberly Wyatt, Ali Rowhani-Rahbar

**Affiliations:** 1 Center for Firearm Injury Prevention, Department of Pediatrics, School of Medicine, University of Washington, 3980 15th Ave NE, Seattle, WA 98195 USA; 2 Department of Epidemiology, School of Public Health, University of Washington, Seattle, WA USA; 3 King County Prosecuting Attorney’s Office, Seattle, WA USA

**Keywords:** Extreme risk protection orders, Behavioral health evaluations, Firearm injury prevention, Judicial decision-making

## Abstract

**Background:**

Extreme Risk Protection Orders (ERPOs) are civil court orders that temporarily restrict firearm access for people at risk of harming themselves or others. In some cases, courts mandate respondents to complete a behavioral health evaluation (BHE), which may create an opportunity to connect people with unmet behavioral health needs to behavioral health services. However, little is known about how often these evaluations are ordered, what factors shape these decisions, or how judges explain their reasoning when a BHE is not ordered. This mixed-methods study examined patterns in court-ordered BHEs among granted ERPOs in Washington State from 2017–2023.

**Methods:**

Quantitative analyses examined how often BHEs were ordered, whether orders varied by respondent or case characteristics, and the frequency of BHE orders over time. We also conducted a qualitative content analysis of judges’ written explanations in cases where an evaluation was considered appropriate but not ordered, using an inductive coding approach to identify common themes.

**Results:**

Between 2017 and 2023, BHEs were ordered in less than half of granted ERPOs. Rurality was significantly (p < 0.05) associated with differences in BHE orders, but differences in BHE orders among other respondent or case characteristics were also observed. BHE orders varied per year, with more than half (55.8%) of ERPOs ordering a BHE in 2023. Judges most often cited recent or ongoing treatment, recent involuntary psychiatric holds, or parallel criminal proceedings as reasons for not ordering an evaluation. Other explanations included procedural considerations, reliance on family supports, and limited ability to monitor compliance.

**Conclusion:**

Findings indicated that BHEs are not consistently used in ERPO cases in Washington State, and their use varies by respondent and case characteristics, geography, and service access. Addressing gaps in statutory clarity, strengthening systems for monitoring compliance, supporting coordination across legal systems, and expanding access to affordable, voluntary behavioral health services may help ensure that ERPOs more consistently support both firearm safety and connection to appropriate care.

**Supplementary Information:**

The online version contains supplementary material available at https://doi.org/10.1186/s40621-026-00698-x.

## Background

Extreme Risk Protection Orders (ERPOs) are civil orders designed to reduce firearm-related harm by temporarily removing and restricting firearm access for individuals who are at substantial risk of harming themselves or others [1]. As of January 2026, 22 states, the District of Columbia, and the U.S. Virgin Islands have adopted ERPO policies, also referred to as gun violence restraining orders or “red-flag laws” [2, 3]. Evidence suggests that ERPOs are effective in reducing suicides and are also used to prevent mass shootings, intimate partner violence, and firearm homicides [4–6].

In Washington State, the ERPO law went into effect in December 2016 [7]. Eligible petitioners include law enforcement, intimate partners, and family or household members [8]. Once an ERPO petition is filed, during or after court hours, a judge will determine if there is a legal basis to grant a temporary order; if granted, the temporary order will be in place for up to 14 days at which time the court will determine whether a full order should be granted or the temporary order should be continued for another fixed period of time (e.g. to effectuate personal service, or continued by agreement of the parties) [7, 9, 10]. If granted, the full ERPO usually lasts for one year; however, respondents may petition the court for early termination prior to the expiration of the ERPO. Both the temporary and full ERPO requires the respondent to immediately surrender their firearms and concealed pistol licenses to law enforcement. Within 90 days of expiration, the petitioner may file a motion with the court to renew the ERPO for an additional year. The ERPO respondent will be served with the motion to renew and will have an opportunity to appear in court and be heard. When an ERPO is granted at the full order hearing, the judge may also determine whether a behavioral health evaluation (BHE) (e.g., mental health or chemical dependency evaluation) is appropriate and order the respondent to complete the evaluation within a specified timeframe while the ERPO is in effect [7]. The ERPO statute (Initiative 1491) states that “the court shall consider whether a mental health evaluation or chemical dependency evaluation is appropriate, and may order such evaluation if appropriate” [7]. While the statute states that the ERPO must indicate whether a BHE is required, the statute does not specify a timeframe for completion or who is responsible for covering associated costs and monitoring compliance. Therefore, respondents may be responsible for locating and paying for the evaluation.

Because ERPOs are often sought when respondents are demonstrating high-risk behaviors, ERPOs may create opportunities to connect respondents—many of whom are experiencing behavioral health concerns but may not be actively receiving services [11]—with needed support. Thus, while ERPOs were not designed as a behavioral health intervention, ERPOs may nonetheless serve as a pathway to behavioral health support. Current research indicates that ERPOs are often underutilized in this capacity, despite many civilian petitioners citing a desire to connect respondents to behavioral health services as one of the motivating factors for filing the ERPO petition [12]. For example, in Colorado, over one-third of ERPO petitions filed over a three-year period referenced respondents’ behavioral health concerns, yet only 5% of those respondents were court-ordered to complete a BHE [13].

Judicial decisions about BHEs may be informed by characteristics and behaviors of respondents as well as contextual and systems-level factors, including respondents’ behaviors during the precipitating event(s) (i.e., the incident(s) leading to ERPO filing), prior criminal history, prior civil orders (including previous ERPOs and domestic violence protection orders), and documented behavioral health concerns, along with behavioral health service capacity [10]. However, since Washington State’s ERPO statute does not provide specific guidance on ordering BHEs, there may be variability in how frequently and under what circumstances judges order such evaluations. Indeed, prior research indicates judges’ sentencing decisions in criminal cases can be influenced by the availability of behavioral health resources in their jurisdiction [14], suggesting similar dynamics may affect decisions in ERPO cases.

This study sought to determine how frequently BHEs were ordered among granted full ERPOs in Washington State over time, describe respondent and case characteristics among those with and without a court-ordered BHE, and analyze judicial justifications when indicated, among full order ERPOs where BHEs were deemed appropriate, but not court ordered.

## Methods

### Study sample and data abstraction

This study included ERPO cases granted at the full order hearing from December 2016 through December 2023. We systematically reviewed, abstracted, and coded all petitions and associated court records for these cases. Each coder (NA, RR, AM) reviewed the complete set of court documents and abstracted relevant data for entry into Research Electronic Data Capture Database (REDCap), a secure web-based data management system [15, 16]. To ensure consistency and reliability in the abstraction process, cases were double coded until inter-rater reliability reached 0.80, as described in previous work [6, 17]. The research team additionally met regularly to discuss coding procedures and resolve any discrepancies.

### Variables

The primary variable of interest was whether a judge ordered a BHE to be completed by the respondent at the full order hearing. This variable was derived from the court forms, which allowed judges to select one of three options regarding the appropriateness of a BHE: (1) ‘Appropriate,’ (2) ‘Not Appropriate,’ or (3) ‘While appropriate, the court is not ordering an evaluation for the following reason(s)’ [see Additional Fig. 1]. Options (1) and (2) are presented as checkboxes on the full order form, while option (3) includes checkbox and an accompanying open text field, allowing judges to provide additional justification, if necessary. We dichotomized this variable, as either ordered or not ordered. Evaluations were classified as ordered if the abstracted BHE variable was marked as appropriate and included a specified timeframe for completion. Cases abstracted as not appropriate, appropriate but not ordered, or left unmarked were classified as not ordered because no court-ordered BHE requirements were documented. We excluded 11 cases in which a BHE was designated as appropriate, but lacked sufficient documentation to determine whether an evaluation was ordered. Respondents with multiple ERPOs during the study period were classified as having been ordered to complete a BHE if at least one of their ERPOs mandated the completion of a BHE.

Respondent characteristics included sex, race/ethnicity, age, and veteran status. Case characteristics included petitioner type, documented threats of harm, county rurality (based on Rural–Urban Commuting Area codes for the county in which the ERPO was filed), any history of behavioral health concerns indicated in the petition, behavioral health concerns occurring during the precipitating event, and state behavioral health proceedings occurring during the precipitating event (i.e., involuntary behavioral health treatment). Petitioners were categorized as family or household members (non-intimate partners), intimate partners, or law enforcement. The type of threats of harm made during the precipitating event included threats of harm to self, others, or both. County rural–urban designation was also included as a proxy for the availability of behavioral health resources, drawing on prior research indicating that rural counties generally have fewer behavioral health providers per capita [18].

### Data analysis

Descriptive statistics were calculated using R version 4.5.1 to examine the distribution of respondent and case characteristics among ERPOs granted at the full order hearing, stratified by the dichotomous BHE variable (e.g., ordered/not ordered). We conducted chi-squared tests to assess whether there were significant differences (at alpha 0.05) in BHE orders by respondent and case characteristics. We further examined changes over time by calculating, for each year, the proportion of granted ERPOs that ordered a BHE.

We also conducted a qualitative content analysis among ERPO cases where the judge marked the BHE as “while appropriate, the court is not ordering an evaluation for the following reason(s).” The justification provided in the open-text field was used to assess judicial reasoning. An inductive approach was used to systematically identify recurring words, phrases, and concepts to develop descriptive categories that captured the range of judicial reasoning; because individual justifications often contained multiple elements, these categories were not mutually exclusive. We then condensed these categories into broader themes reflecting common patterns. Representative examples from the justifications were mapped to each theme to show how they were applied in practice. This analytic process aligned with methodological guidance for qualitative content analysis [19, 20].

## Results

There were 957 granted full ERPOs between December 2016 and December 2023. Among granted full ERPOs with complete information about BHEs (N = 946), a BHE was marked as appropriate and ordered in 383 (40.5%) cases. Of the unique respondents with complete information about the BHE ordered at the full order hearing (N = 931), 380 (40.8%) were mandated to complete a BHE for at least one ERPO. Among cases where a BHE was not ordered (n = 563), 141 (25.0%) were marked as appropriate but not ordered, 97 (17.2%) were marked as not appropriate, and 325 (57.7%) cases were left unmarked and were classified as not appropriate and not ordered.

### ERPO respondent characteristics

**Fig. 1:**
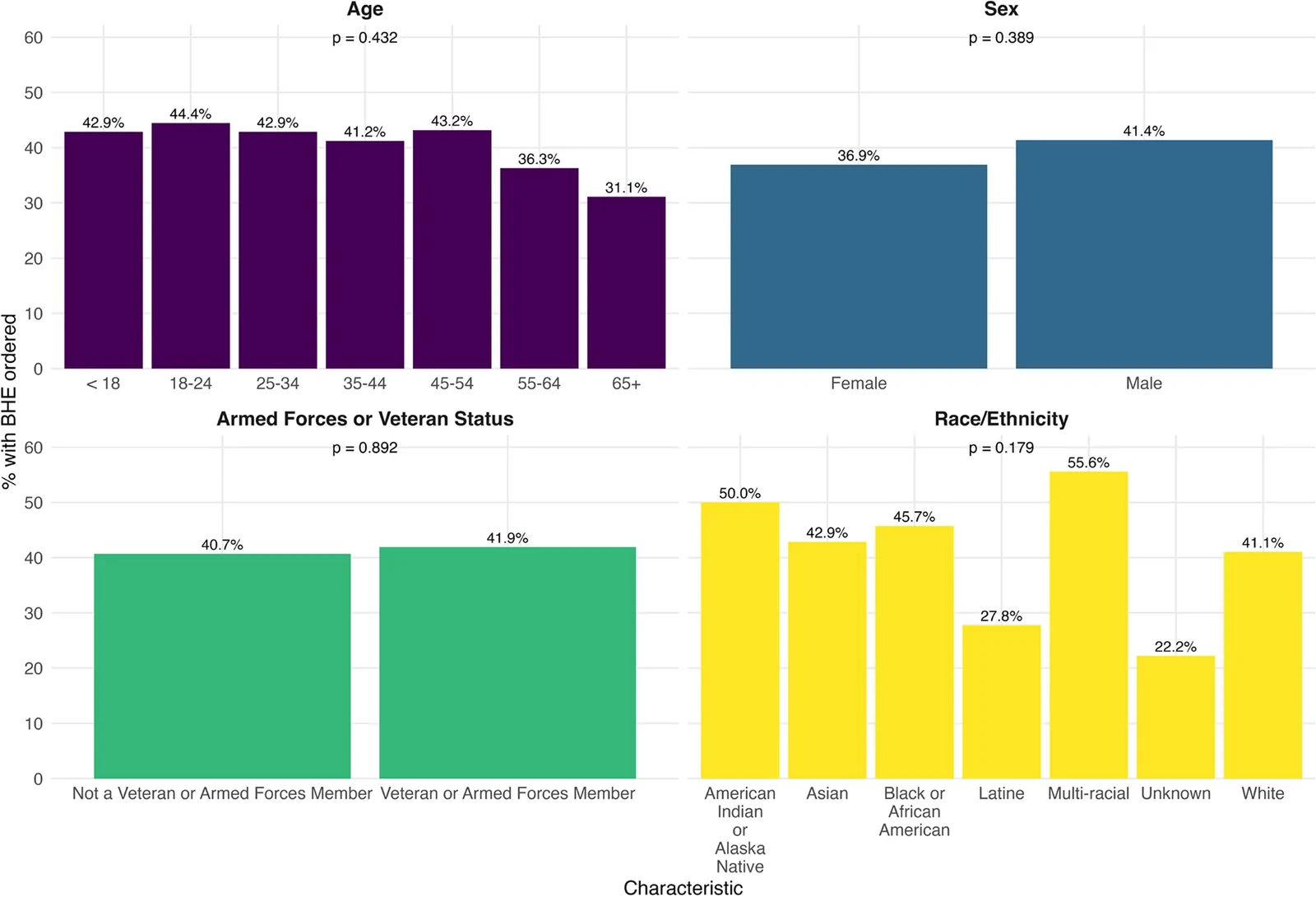
Proportion of respondents with BHE order, by respondent characteristic

Among the entire sample of respondents, most respondents were identified in court documents as male (86.0%), White (76.1%), 25–44 years old (49.0%), and not a veteran or military member (88.7%) (Table 1). Within each demographic subgroup, BHEs were most often ordered among respondents who were aged 18–24 years old (44.4%), male (41.4%), multi-racial (55.6%), and Veterans or military members (41.9%) (Fig. 1). No significant associations were identified between respondent characteristics and BHE orders.

**Table 1:** Respondent characteristics (N = 931)

**Respondent characteristics**	**N (%)**
**Sex**	
Female	130 (14.0%)
Male	800 (86.0%)
Missing	NA
**Race/ethnicity**	
American Indian or Alaska Native	6 (0.6%)
Asian	49 (5.3%)
Black or African American	94 (10.1%)
Latine	18 (1.9%)
Multi-racial	18 (1.9%)
Native Hawaiian or Pacific Islander	NA
Other	NA
Unknown	36 (3.9%)
White	704 (76.1%)
Missing	NA
**Age**	
< 18	7 (0.8%)
18–24	126 (13.5%)
25–34	245 (26.3%)
35–44	211 (22.7%)
45–54	139 (14.9%)
55–64	113 (12.1%)
65 +	90 (9.7%)
**Veteran or armed forces status**	
Not a Veteran or Armed Forces Member	826 (88.7%)
Veteran or Armed Forces Member	105 (11.3%)

Cells < 5 or that were missing are suppressed

### ERPO case characteristics

Among all ERPO cases, the most frequent type of threat documented in the petition was threats of harm to others only (49.8%), followed by threats of harm to self only (27.1%) and threats of harm to both self and others (23.1%) (Table 2). Law enforcement filed most petitions (93.0%), and 85.0% of cases occurred in an urban county. More than three-quarters of cases (84.6%) documented any history of behavioral health concerns, and 56.0% of cases indicated behavioral health concerns occurring during the precipitating event.

**Table 2:** Case characteristics (n = 946)

**Case characteristic**	**N (%)**
**Threat type**	
Harm to others	471 (49.8%)
Harm to self	256 (27.1%)
Harm to self and others	219 (23.2%)
**Petitioner type**	
Civilian	66 (7.0%)
Law enforcement	880 (93.0%)
**County rurality**	
Rural	142 (15.0%)
Urban	804 (85.0%)
**History of behavioral health concerns**	
No	129 (13.6%)
Yes	817 (86.4%)
**Behavioral health concerns during the precipitating event**	
No	415 (43.9%)
Yes	531 (56.1%)
**Behavioral health proceedings during precipitating event**	
No	648 (68.5%)
Yes	298 (31.5%)
**Documented compliance of behavioral health evaluation**	
No	27 (2.9%)
Yes	42 (4.4%)
Missing	877 (92.7%)

**Fig. 2:**
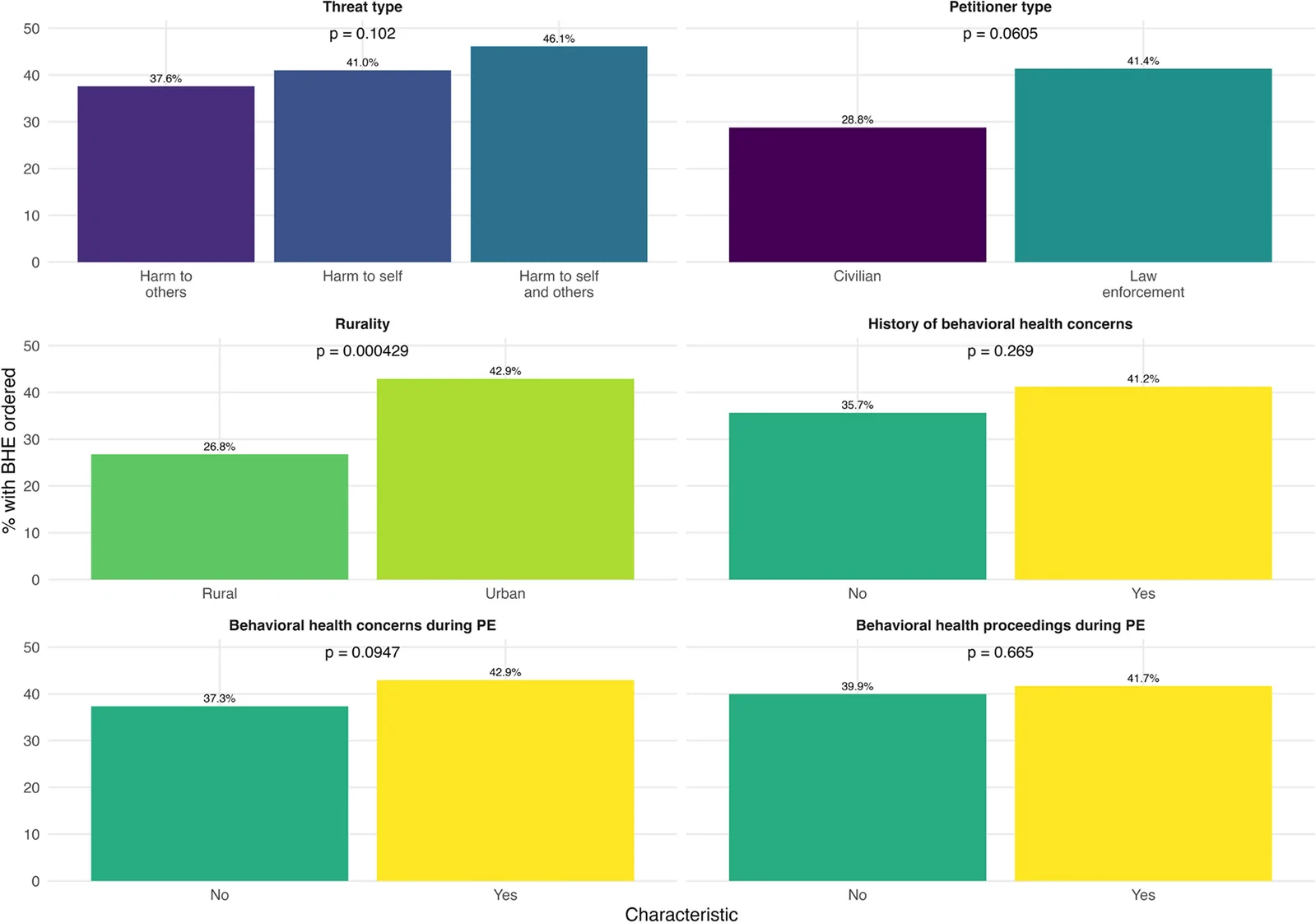
Proportion of cases with BHE order, by case characteristic. *PE: precipitating Event*

BHEs were most commonly ordered among cases documenting threats of harm to self and others (46.1%), those with law enforcement petitioners (41.4%), those occurring in urban counties (42.9%), and those documenting a history of behavioral health concerns (41.2%) or behavioral health concerns occurring during the precipitating event (42.9%) (Fig. 2). Of respondents who were subject to state behavioral health proceedings (e.g., evaluation or detention under Washington State’s Involuntary Treatment Act) during the precipitating event, 41.6% were ordered a BHE. County rurality (χ^2^ p < 0.001) was significantly associated with BHE orders, with a higher proportion of evaluations ordered in urban counties compared to rural counties (42.9% vs 26.8%).

The majority (92.8%) of cases with a court-ordered BHE did not have any information on whether a BHE was completed or whether the respondent was in compliance with this specific provision of the court order. Among cases with complete information about BHE compliance (n = 69), 42 (60.9%) showed that the respondent did complete a BHE.

### Trends in BHEs over time

**Fig. 3:**
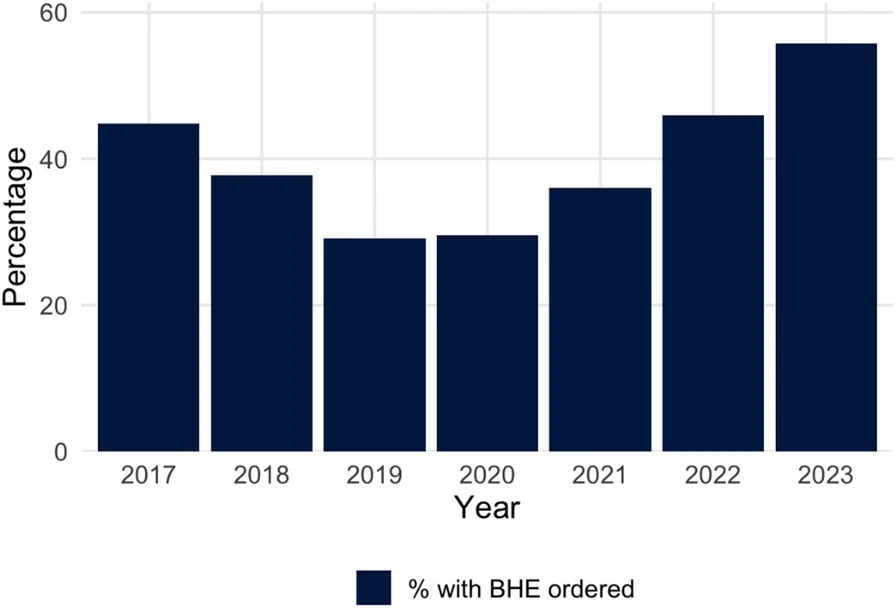
Percent of granted ERPOs with behavioral health evaluations ordered by year, 2017–2023

Between 2017 and 2023, the proportion of ERPOs that included a court-ordered BHE varied annually, ranging from 29.1% in 2019 to 55.8% in 2023 (Fig. 3).

### Content analysis

Among the 141 ERPO cases marked “while appropriate, BHE not ordered,” petitions most frequently documented threats of harm to others (n = 68, 48.2%), followed by harm to self (n = 47, 33.3%) and both self and others (n = 26, 18.4%). In most of these cases (n = 122, 86.5%), the petition referenced behavioral health concerns occurring during the precipitating event [see Additional Table 1]. The analysis revealed six primary themes in judicial reasoning that emphasized pre-existing care, reliance on other systems, and structural or procedural gaps perceived to impact enforcement: 1) existing or recent voluntary treatment or evaluation, 2) pending or ongoing criminal proceedings, 3) recent involuntary care or commitment, 4) procedural conditions and ambiguity, 5) deferred to informal supports, and 6) enforceability limitations and barriers to access (Table 3).

**Table 3:** Themes in judicial justifications for not ordering behavioral health evaluations, even when appropriate (N = 141)

**Theme**	**N (%)**	**Description**	**Example(s)**	**Interpretation**
*Existing or recent voluntary treatment/evaluation*	*73 (51.8%)*	Respondent is currently engaged in voluntary outpatient treatment or had recently completed an evaluation	“Respondent is currently in treatment with the VA.” “Respondent has already engaged in therapy for alcohol, PTSD.”	Judges viewed existing or recent voluntary care as sufficient
*Pending/ongoing criminal proceedings*	*35 (24.8%)*	Evaluation expected through parallel criminal legal processes	“Pending criminal case where this issue will be addressed.” “Respondent will be entering a plea agreement which requires his participation in the mental health treatment.”	Courts deferred to criminal legal proceedings
*Recent involuntary care or civil commitment*	*14 (9.9%)*	Respondent had recently undergone involuntary treatment/hospitalization or psychiatric hold	“Police reports indicate recent mental health ITA hold.” "Respondent just completed 15 days inpatient psychiatric treatment at a hospital."	Involuntary care was noted as a substitute for ordering another evaluation
*Procedural conditions & ambiguity*	*13 (9.2%)*	Procedural circumstances or evaluation are not requested by the petitioner. Statute does not provide specific guidance	"While appropriate, the respondent may obtain an evaluation in order to request a termination of this order." “Not requested.”	Judicial discretion may be impacted by statutory ambiguity
*Deferred to informal supports*	*6 (4.3%)*	Judges cited reliance on family or caregivers for decision making	“Family dealing with these issues.” “Stipulation and intervention by family.”	Courts deferred to personal support when deemed sufficient
*Enforceability limitations & access barriers*	*6 (4.3%)*	Judges cited a lack of mechanisms to track or enforce compliance and a lack of available services to care	“No way to monitor.” “Respondent may not have resources.”	Judicial discretion is constrained by monitoring and resource limitations

These themes are not mutually exclusive. Therefore, percentages may exceed 100%

### Existing or recent voluntary treatment/evaluation

The most common justification (n = 73, 51.8%) involved respondents who were already receiving voluntary behavioral health treatment or could provide proof that they had recently undergone an evaluation. Judges referenced ongoing outpatient therapy (e.g., treatment through the Veteran Affairs care network) as adequate to address the respondent’s behavioral health concerns, making a court-ordered evaluation redundant. This rationale was particularly common in ERPO cases where threats of harm to self were the primary concern during the precipitating event (n = 47); in these cases, 70.2% (n = 33) of justifications noted existing treatment or recent evaluation. Similarly, in cases involving threats to both self and others (n = 26), 53.8% (n = 14) referenced ongoing, voluntary care when determining that no additional BHE was required. Across cases, judges did not provide a temporal definition for what constituted a “recent” treatment or evaluation.

### Pending/ongoing criminal proceedings

In 24.8% of cases (n = 35), judges deferred to ongoing criminal legal proceedings where behavioral health needs were expected to be addressed through sentencing conditions, plea agreements, or court-ordered treatment, particularly when judges noted concerns about how a BHE may impact the respondent’s Fifth Amendment right to prevent self-incrimination. In cases with documented threats of harm to others (n = 68), judges most frequently deferred to pending or ongoing criminal proceedings for decision-making surrounding court-ordered behavioral health interventions (n = 26, 38.2%). For example, some justifications noted that the respondent’s participation in treatment was already a condition of the plea agreement, highlighting that judicial decision-making in ERPO cases may intersect with criminal legal contexts.

### Recent involuntary care or civil commitment

In 9.9% of cases (n = 14) the respondent had recently experienced an involuntary psychiatric hold, inpatient stay, or another form of court-ordered care. Judges generally deemed these recent interventions sufficient, determining that another evaluation was duplicative. Like other justification categories, judges did not define the timeframe for what constituted “recent” involuntary care.

### Procedural conditions & ambiguity

Judges described specific procedural circumstances and statutory ambiguity as factors that influenced whether they ordered a BHE (n = 13, 9.2%). Some noted that an evaluation would only be required if the respondent wanted to file a motion to terminate the ERPO, while others reasoned that the petitioner had not explicitly requested a BHE.

### Enforceability limitations & access barriers

Judges noted barriers related to either enforceability or access to resources (n = 6, 4.3%). Some judges mentioned concerns that the court lacked the ability to monitor compliance once a BHE was ordered, while others noted the respondent’s lack of access to the appropriate resources to comply with a court-ordered evaluation. Of these cases, more than half occurred in rural counties.

### Deferred to informal supports

In some cases, judges deferred to existing informal support (e.g., family members or caregivers) in managing behavioral health concerns, rather than ordering a formal evaluation (n = 6, 4.3%); almost all of these cases involved documented threats of harm to self.

## Discussion

This study describes how often and under what circumstances BHEs are ordered as part of ERPO adjudication in Washington State and provides insights into judicial decision-making, an element not captured in existing literature. This study highlights potential disparities in the ordering of these evaluations, identifies gaps in current ERPO implementation, and explores the underlying reasons for variation in BHE orders. To our knowledge, this is the first study to integrate both quantitative and qualitative approaches to examine ERPO implementation in relation to BHE orders.

Both our quantitative and qualitative analyses demonstrated a disparity in respondents’ connection to BHEs in rural counties. Respondents living in urban counties were significantly more likely to be ordered to complete a BHE than those in rural counties. However, this finding could reflect differences in ordering practices rather than underlying need, which we did not assess. Additionally, judges in rural counties frequently cited concerns about respondents’ ability to access resources needed to comply with a BHE, opting not to impose the requirement to avoid undue burdens. Prior work has established that the supply of behavioral healthcare providers in nonmetropolitan areas is significantly lower than in more urban areas, particularly for certain types of treatment such as outpatient substance use treatment or psychiatry [18, 21]. Rural respondents may face additional barriers related to affordability and acceptability [22]. These findings raise important questions about whether mandating BHEs for rural respondents may fail to address underlying behavioral health needs when completing a BHE is not feasible and may contribute to noncompliance, which could result in the renewal of the ERPO beyond the initial one-year period and extend respondents’ involvements with the civil legal system. This suggests that future policy considerations should weigh the benefits of BHE requirements against the practical realities of resource availability and implement strategies to ensure equitable access to care.

Notably, our quantitative analyses highlighted that less than half of cases that cited behavioral health concerns occurring during the precipitating event were ordered to complete a BHE. This finding is important as behavioral health concerns occurring during the precipitating event may indicate an acute need for intervention, yet the qualitative analysis emphasized that procedural and statutory considerations—such as deferral to recent treatment, involuntary commitments, or parallel criminal proceedings—may influence the decision to not order a BHE rather than the absence of behavioral health concerns.

Judges explicitly cited recent involuntary psychiatric holds or civil commitments as reasons not to order a BHE at the full order hearing, even though what constituted “recent” is not clearly defined. In some cases, respondents may have also been transported by law enforcement to the hospital for evaluation related to Involuntary Treatment Act (ITA) proceedings during the precipitating event, which may have influenced judicial perceptions regarding the need for an additional court-ordered BHE. Judges appeared to view these prior assessments as sufficient for ERPO purposes, allowing the respondents to rely on prior evaluations rather than requiring completion of an additional court-ordered evaluation. Although relatively little is known about the potential benefits of court-ordered BHEs in the ERPO context, existing research suggests that involuntary mental health holds do not lead to long-term improvements in mental health outcomes and may even reduce trust in mental health care systems and decrease the likelihood of future help-seeking behaviors by people in crisis [23]. In addition, involuntary treatment has been viewed as a form of coercion, leaving individuals with diminished autonomy over their care and self-reported dissatisfaction with treatment [24]. These findings suggest that there are limitations of relying on involuntary commitments or court-ordered BHEs alone to address the behavioral health needs of respondents and it does not necessarily guarantee continuity of care after release.

The most common justification for not ordering a BHE at the full order hearing involved respondents who were already engaged in treatment or had recently undergone an evaluation. This deferral suggests an effort to avoid redundancy and prioritize ongoing care. However, it also raises the possibility of under-evaluating respondents’ circumstances, particularly given that behavioral health needs are dynamic and there are limitations in the courts’ ability to communicate with existing providers. Without reassessment, opportunities for intervention adaptations may be missed. In other cases, judges deferred to informal support, such as family members or caregivers, to address the respondent’s behavioral health needs. This suggests that there was evidence that the respondent was adequately supported by those around them and that the respondent’s support system was privy to circumstances surrounding the ERPO process. However, future research should directly assess these supports and examine the actual needs and capacity of family members or caregivers involved in the ERPO process.

Judges also noted their decision to defer the BHE to be addressed by the criminal legal system. In these cases, ERPO respondents had parallel criminal proceedings, and the judges reasoned that the behavioral health concerns would be addressed through sentencing conditions or plea agreements. While this may also help to avoid redundancy, it may be insufficient. Research on behavioral health services delivered through the criminal legal system, such as mental health courts and diversion programs, show variability in the availability, quality, and timeliness of behavioral health services and are also dependent on local resources [25]. Access to behavioral health services in criminal legal settings may also differ from the ERPO context, as care can be coordinated through court-connected programs, whereas ERPO-related BHEs may require respondents to independently locate and pay for services. Additional studies have found that gaps in access to care persist even when treatment requirements are embedded in plea agreements and that individuals involved in the criminal legal system often have unmet behavioral health needs [26, 27].Collectively, these findings suggests that there are opportunities to better integrate behavioral health services across systems.

Less than 10% of cases reflected procedural conditions or ambiguity in, or interpretations of, the statute, where judges indicated that evaluations were only required if the respondent wished to file a motion to terminate the ERPO or when specifically requested by the petitioner. This theme aligned with the legal concept of *non ultra petitum*, where judges may not decide more than they have been asked to [28]. This suggests that clearer statutory language is needed to ensure alignment with the legislative intent of the statute and reduce missed opportunities to connect respondents to appropriate services. Lastly, resource and monitoring constraints were identified as barriers to enforceability and the court’s ability to monitor compliance when BHEs are ordered. These structural limitations highlight the need not only for statutory clarity but also for additional training opportunities to support judges in applying behavioral health considerations and identifying when evaluations are procedurally appropriate.

We also found that in most cases where BHEs were ordered, there was no evidence in the ERPO court record indicating that the respondent completed the evaluation or complied with the order. It is likely that this underestimates actual compliance, as BHEs may occur after the full order hearing and outside the period of which we accessed court documents. Additionally, BHEs are medical records and may be sealed or otherwise inaccessible in publicly available ERPO files due to HIPAA protections. Among respondents whose records did include information on BHE compliance, just over half had completed the evaluation. Respondents may not be able to comply with BHE orders due to barriers to accessing treatment, limited financial resources, or insufficient guidance from the court regarding what constitutes a BHE. Since ERPOs are civil proceedings, most respondents are not represented by an attorney, making a court-provided explanation of the BHE process especially important. Additionally, mental health providers may lack a standardized protocol for conducting these evaluations in the ERPO context, contributing to variability in how BHEs are performed and documented. Courts may choose to grant an ERPO renewal petition if they cannot verify that a BHE has been completed. 

Taken together, these findings suggest that court-ordered BHEs are an important mechanism for identifying behavioral health needs during periods of acute crisis, but prior research suggests that their effectiveness depends on whether respondents choose to access or can access and engage with care. Without adequate infrastructure to support timely referrals, navigation, and follow-through, BHE orders may not translate into meaningful connections to services. These findings point to the need for clearer statutory and judicial guidance on ordering BHEs, alongside practical supports such as care coordination or navigation services, formal referral pathways between courts and community behavioral health providers, and mechanisms to address cost and access barriers to improve continuity of care, reduce reliance on families to manage complex needs, and support respondents’ ability to comply once an order is issued.

### Implications

These findings highlight several areas where ERPO implementation could be strengthened to connect respondents to behavioral health services. First, revisions to statutory language and frameworks are needed to improve clarity and help guide how BHEs are ordered across jurisdictions while also preserving judicial discretion. Second, improvements to infrastructure for monitoring compliance with court-ordered evaluations are needed. Without mechanisms in place to confirm whether respondents comply with court-ordered evaluations, judges may be hesitant to order them, leading to missed opportunities to direct respondents to appropriate care. If monitoring ability is improved, investments in behavioral health services are also needed to reduce access barriers related to availability and cost. At the same time, given evidence that involuntary mental health interventions can limit autonomy and reduce ongoing engagement with behavioral health services due to diminished trust in care, efforts to strengthen ERPO implementation should be paired with investments in voluntary behavioral health services that are accessible, affordable, and culturally responsive. Finally, coordination between civil and criminal legal systems could help avoid gaps in care for respondents with parallel proceedings. Strengthening communication between courts may ensure that behavioral health needs identified in ERPO proceedings are addressed in a timely manner. Collectively, these implications point to the potential for ERPOs to be more consistently leveraged to improve both firearm safety and behavioral health outcomes.

### Limitations

Some limitations should also be noted. First, the analysis relied on brief written justifications, which may not fully reflect judges’ entire decision-making process. Second, many granted ERPO cases were not clearly marked to indicate whether a BHE was ordered (n = 325) and were ultimately categorized as if a BHE was not ordered. This may reflect ambiguity in judicial documentation practices, and could have resulted in misclassification of respondents into BHE groups. Third, because this study focused on Washington State, findings may not be generalizable to other states with different statutes, ERPO processes or judicial practices. Additionally, this study included ERPOs granted during the COVID-19 pandemic, a period when BHEs may have been ordered less frequently due to disruptions in behavioral health services and reduced access to providers. Lastly, while the content analysis provides valuable insights into judicial reasoning, it does not capture the perspectives of other stakeholders, such as petitioners, respondents, legal counsel, or behavioral health providers, which could provide additional insights. Future research that prioritizes interviews with different stakeholders may help address this limitation by contextualizing judicial decision-making, examining barriers to completing BHEs, and assessing how often respondents seek early termination of ERPOs or provide subsequent documentation of completed BHEs.

## Conclusion

ERPOs are designed to reduce firearm-related harm for those who pose a substantial risk to self or others, but they may also serve as a pathway to connect respondents to needed behavioral health services. Our findings reveal potential disparities in the ordering of BHEs, particularly in rural areas where limited provider accessibility may make these evaluations harder to obtain. This study suggests that judges do not always order BHEs even when deemed appropriate, citing existing care, pending criminal proceedings, recent involuntary treatment, reliance on informal supports, and procedural ambiguity or enforceability limitations as justifications for these decisions. Addressing barriers by refining statutory clarity, expanding behavioral health resources, and improving cross-system coordination may enhance the ability to use ERPOs as a tool to promote both firearm safety and positive behavioral health outcomes.

## Supplementary Information

Additional file 1.

## Data Availability

Study data may be available upon request to the corresponding author (Rachel Ross, [rross4@uw.edu]) on reasonable request pending IRB approval.

## Abbreviations

ERPO: Extreme risk protection order
BHE: Behavioral health evaluation
REDCap: Research electronic data capture database
HIPAA: Health insurance portability and accountability act
ITA: Involuntary treatment act
