# Supplementary material for: Patterns of court-ordered behavioral health evaluations among extreme risk protection order respondents in Washington State
Source: Inj Epidemiol. 2026 Jul 10;13:65. doi: 10.1186/s40621-026-00698-x (PMC13637203; doi:10.1186/s40621-026-00698-x)
Supplement: Supplementary file 1 [file 40621_2026_698_MOESM1_ESM.docx]

Additional File 1

Patterns of Court-Ordered Behavioral Health Evaluations among Extreme Risk Protection Order Respondents in Washington State

Rachel Ross, MPH (rross4@uw.edu),^a^ Erika Marts, MPH (ekmarts@uw.edu),^a,b^ Nicole Asa, MPH (nasa3@uw.edu),^a,b^ Samantha Banks, MS (sbanks94@uw.edu),^a^ Kelsey M. Conrick, PhD, MPH (kmc621@uw.edu),^a^ Esprene Liddell-Quintyn, PhD (esprene@uw.edu),^a^ Ayah Mustafa, BS (mustafaa@uw.edu),^a^ Sandra Shanahan (sandra.shanahan@kingcounty.gov),^c^ Julia P.Schleimer, PhD, MPH (jps36@uw.edu),^a^ Kimberly Wyatt (kimberly.wyatt@kingcounty.gov),^c^ Ali Rowhani-Rahbar, MD, PhD, MPH (rowhani@uw.edu)^a,b^

^a^Center for Firearm Injury Prevention, Department of Pediatrics, School of Medicine, University of Washington, Seattle, WA, USA

^b^Department of Epidemiology, School of Public Health, University of Washington, Seattle, WA, USA

^c^King County Prosecuting Attorney’s Office, Seattle, WA, USA

Additional Figure 1. Final ERPO Order Excerpt Documenting Behavioral Health Evaluation Decision

Additional Table 1. Most Common Judicial Justifications Themes by Threat Type

| **Threat Type** | **Total cases (n)** | **Most Common Justification Themes, n (%)** |
| --- | --- | --- |
| Harm to Self | 47 | Existing or recent treatment/evaluation (33, 70.2%); informal supports (5, 10.6%); procedural ambiguity (5, 10.6%). |
| Harm to Others | 68 | Pending/ongoing criminal proceedings (26, 38.2%); Existing or recent treatment/evaluation (21, 30.8%); Recent involuntary commitment (8, 11.7%) procedural ambiguity (6, 8.8%). |
| Harm to Both | 26 | Existing or recent treatment (14, 53.8%); Pending/ongoing criminal proceedings (7, 26.9%); Recent involuntary commitment (4, 15.4%). |
